# Preventing Prolonged Times to Awakening While Mitigating the Risk of Patient Awareness: Gas Man Computer Simulations of Sevoflurane Consumption From Brief, High Fresh Gas Flow Before the End of Surgery

**DOI:** 10.7759/cureus.55626

**Published:** 2024-03-06

**Authors:** Franklin Dexter, Richard H Epstein, Anil A Marian, Carlos E Guerra-Londono

**Affiliations:** 1 Anesthesia, University of Iowa, Iowa City, USA; 2 Anesthesiology, Perioperative Medicine, and Pain Management, University of Miami Miller School of Medicine, Miami, USA; 3 Anesthesiology, Perioperative Medicine, and Pain Management, Henry Ford Health System, Detroit, USA

**Keywords:** global warming, case duration, sevoflurane, inhalational anesthesia, volatile anesthetic, pharmacokinetics (pk), computer simulation, management science, hospital engineering, industrial engineering

## Abstract

Prolonged times to tracheal extubation are associated with adverse patient and economic outcomes. We simulated awakening patients from sevoflurane after long-duration surgery at 2% end-tidal concentration, 1.0 minimum alveolar concentration (MAC) in a 40-year-old. Our end-of-surgery target was 0.5 MAC, the Michigan Awareness Control Study's threshold for intraoperative alerts. Consider an anesthetist who uses a 1 liter/minute gas flow until surgery ends. During surgical closure, the inspired sevoflurane concentration is reduced from 2.05% to 0.62% (i.e., MAC-awake). The estimated time to reach 0.5 MAC is 28 minutes. From a previous study, 28 minutes exceeded ≥95% of surgical closure times for all 244 distinct surgical procedures (N=23,343 cases). Alternatively, the anesthetist uses 8 liters/minute gas flow with the vaporizer at MAC-awake for 1.8 minutes, which reduces the end-tidal concentration to 0.5 MAC. The anesthetist then increases the vaporizer to keep end-tidal 0.5 MAC until the surgery ends. An additional simulation shows that, compared with simulated end-tidal agent feedback control, this approach consumed 0.45 mL extra agent. Simulation results are the same for an 80-year-old patient. The extra 0.45 mL has a global warming potential comparable to driving 26 seconds at 40 kilometers (25 miles) per hour, comparable to route modification to avoid potential roadway hazards.

## Introduction

In the current clinical setting, anesthetists are expected to follow safe, efficient, and sustainable anesthesia practices. An ideal example of this balance is to consider the processes to achieve prompt awakening of patients from sevoflurane anesthesia while using low fresh gas flows. Maintaining a relatively high inspired concentration until surgery is finished and only then turning off the vaporizer and increasing fresh gas flows can result in long times to awakening. Prolonged times to tracheal extubation (≥15 minutes) [1,2] are associated with risk of reintubation [3], respiratory treatments in the post-anesthesia care unit [3], the administration of flumazenil and naloxone [3], poor-quality ratings [4], longer times in the operating room [5], and non-anesthesia practitioners in the operating room waiting idly for extubation [6].

One approach to achieve a target end-tidal concentration at the end of surgery is to turn the vaporizer off early. However, an anesthetist could forget to turn the vaporizer back on [7] if distracted and the closure takes longer than expected. (By "anesthetist," we mean the practitioner continually with the patient, adjusting the vaporizer and fresh gas flows.) Closure times have large proportional variability, even greater than surgical times [8]. Times remaining in surgical cases do not decrease like simple countdown timers [8-10]. Therefore, in the current study, we simulated several scenarios of the alternative approach of decreasing the inspired agent concentration but not turning the vaporizer off, with or without increasing the fresh gas flows. Our goals were estimation of the resulting cost in extra milliliters of sevoflurane consumed and comparisons of the times to achieve a pre-specified end-tidal concentration target.

## Materials and methods

We used Gas Man, Version 4.3.6227 Professional (Med Man Simulations, Inc., Chestnut Hill, MA), with selection of its default options (e.g., cardiac output) [11]. Gas Man more accurately predicts end-tidal sevoflurane in oxygen and air than target-controlled infusion pumps achieve desired effect site concentrations of propofol [11]. Gas Man has the parameters of a physiological model [12] but acts like a multicompartment model [13]. Because its results are analytical solutions to systems of differential equations, the same values are obtained by anyone using the same settings (i.e., no standard errors or confidence intervals need to be reported). Philips previously reviewed this screen-based simulator program [12].

Our simulation strategy was broadly the same as applied earlier to examine the probability distribution of times to awakening and their associations with agent consumption [14,15]. Times to achieve decrements in simulated end-tidal concentrations are affected minimally by obesity [16] but are reduced for older patients [17], so we simulated two ages of patients.

For all simulations, we started with 2.05% sevoflurane for 8 hours at 8 liters of fresh gas flow to achieve the steady state of complete simulated uptake of sevoflurane (Table 1), where 2.05% achieves the target of 2% end-tidal, the minimum alveolar concentration (MAC) for 40‑year-olds at one atmosphere [18]. We set a target for the end of surgery of 0.5 MAC, 1% sevoflurane because that was the threshold for intraoperative alerts in the Michigan Awareness Control Study [19]. During simulated closure, we kept the vaporizer ≥0.62%, MAC-awake [18], which is higher than MAC amnesia [20]. Once surgery had been completed and the neuromuscular blockade had been reversed, the vaporizer was turned off fully, and the fresh gas flow was increased. Our simulations were initiated from a steady-state end-tidal concentration (partial pressure) because MAC, MAC-awake, and MAC-aware are based on observed steady-state end-tidal concentrations [18-20], not inferred vessel-rich group concentrations. For our investigation of surgical closure, sevoflurane would not be used for neuromuscular relaxation. Therefore, there is no relevant target for the muscle or fat compartments.

**Table 1:** Simulations #1-3 described in the text ^a^Before the start of closure, the vaporizer sevoflurane setting was 2.05% to achieve an end-tidal concentration of 2.0%, which was continued long enough to reach a steady state. ^b^One of our results is that 28 minutes was the time to achieve an end-tidal sevoflurane concentration of 0.5 minimum alveolar concentration. That duration can be compared with the fifth percentiles and 5% prediction limits of Table 2. ^c^Our second result is the difference of 1.46 mL and 1.01 mL=0.45 mL sevoflurane.

Simulation #	Minutes from the start of closure	Sevoflurane vaporizer (%)	Fresh gas flow (liters per minute)	End-tidal sevoflurane (%)	Sevoflurane delivered (mL)
1	0	0.62^a^	1	2.00^a^	0.00
1	28^b^	0.62	1	1.00	0.95
2	0	0.62^a^	8	2.00^a^	0.00
2	1.8	0.68	1	1.00	0.49
2	28	0.68	1	1.00	1.46^c^
3	0	0.00^a^	8	2.00^a^	0.00
3	0.9	0.68	1	1.00	0.00
3	28	0.68	1	1.00	1.01^c^

The simulated times to achieve the target of 0.5 MAC were compared to data from an earlier epidemiological study of surgical closure times [8]. In Table 2, we summarize fifth percentiles and 5% lower prediction limits for the times from the start of closure to the end of surgery for different surgical procedures, from the earlier study [8]. The specified degree of confidence equals one minus the prediction bound [8,21-23]. Anesthetists' performance relative to surgical time periods needs to be reliable (≥95% success in achieving the objective) despite the very large variability in the times, even when adjusting for the procedure [8‑10,21‑23]. The sample size in Table 2 is greater than the N=23,343 in [8] because, in that study, the combinations used were surgeon and procedure, thereby reducing the sample size. The relevant comparator desired for the current study is combinations of procedures. When sample sizes are reduced, the 5% lower prediction limits become smaller, and, therefore, our desired comparison with the estimated minutes from the simulations is more reliable. In other words, categorizing closure by procedure instead of surgeon and procedure deliberately resulted in larger estimates of 5% prediction limits of times from the start of closure to the end of surgery, increasing the chance of the simulated minutes being sufficiently brief to achieve the MAC target by the end of closure.

**Table 2:** 5% prediction limits and fifth percentiles of time from the start of surgical closure until the end of surgery for the 244 procedures, each with at least 29 cases ^a^For example, the most common procedure was "Laparoscopy, surgical prostatectomy, retropubic radical, including nerve sparing, includes robotic assistance, when performed." The second most common procedure was "Laparoscopy, surgical, gastric restrictive procedure; longitudinal gastrectomy (i.e., sleeve gastrectomy)." ^b^Nominally, the fifth percentile does not apply in practice because each case is new (i.e., what matters is a prediction of the future). For example, when evaluating if an anesthesiologist can give a nurse anesthetist a break for lunch or a breast milk pumping session, during the period from the start of surgery until the end of surgery, what matters is comparing the 5% prediction limit to the duration of the break [21-23]. We included fifth percentiles because surgical times follow log-normal distributions [21], probably less so closing times [9]. We, therefore, wanted to ensure that our results were insensitive to the method of analysis. Yes, they are, as shown by the last two rows of this Table 2.

Category	Result
Cases among Current Procedural Terminology procedures, each with at least 29 cases	29,626
Procedures, each with at least 29 cases	244^a^
Cases per procedure, mean (standard deviation) among the 244 procedures	121 (168)
Mean (standard deviation) among the 244 procedures of the mean minutes from the start of surgical closure to the end of surgery	21.7 (11.3)
Mean (standard deviation) among the 244 procedures of the coefficient of variation of the minutes from the start of surgical closure to the end of surgery	69.8% (21.3%)
Mean (standard deviation) among the 244 procedures of the fifth percentile in minutes, estimated using R-6 percentile method^b^	5.22 (3.68)
Mean (standard deviation) among the 244 procedures of the 5% lower prediction limit based on the log-normal distribution (i.e., takes into account the sample size of cases for the procedure) [21-23]^b^	6.03 (3.97)
Maximum minutes among the 244 procedures of the fifth percentile of the time from the start of closure to the end of surgery, for comparison with 28 minutes needed from Simulation #1	20.0, all <28 minutes
Maximum minutes among the 244 procedures of the 5% prediction limits of the time from the start of closure to the end of surgery, for comparison with 28 minutes needed from Simulation #1	21.2, all <28 minutes

## Results

Simulation #1 considered an environmentally focused anesthetist [24] who uses a 1 liter per minute fresh gas flow until the end of surgery (Table 1). During surgical closure, the inspired concentration of sevoflurane was reduced from 2.05% to 0.62% (i.e., MAC-awake). The end-tidal sevoflurane decreases progressively, functionally following double exponential decay [13,14]. After 28 minutes, the target of end-tidal 1.0% (i.e., 0.5 MAC) is achieved (Table 1).

Among 244 surgical procedures, each with at least 29 cases, the maximum value for the fifth percentiles of the closure time was 20 minutes (Table 2). Thus, the simulated 28 minutes to reach the 1.0% (0.5 MAC) target was too long to be achieved reliably (≥95%) for every procedure. The maximum value for the 5% prediction limits of the closure time was 21 minutes, which was also briefer than 28 minutes (Table 2). Therefore, Simulation #1 shows that, when using low fresh gas flows and sevoflurane for long-duration cases, there will be some prolonged extubations caused by higher than target end-tidal concentration when surgery is done. If the patient were also receiving other anesthetic drugs lowering the suitable target threshold below 1.0% sevoflurane for the end of surgery [25], then the conclusion would still hold because the time to reach the target would be even longer than 28 minutes. In addition, if there were surgical stimulation for portions of surgical closure (e.g., substantive tissue tension when closing fascia), then the conclusion would still apply because the times available to the anesthetist to reduce the agent concentration would be even briefer than given in Table 2.

Suppose instead (Simulation #2) that, during closure, the anesthetist uses a fresh gas flow of 8 liters per minute with the vaporizer at 0.62% until the end-tidal concentration has decreased to 1.0% (Table 1). Reaching this target takes just 1.8 minutes. The anesthetist then increases the vaporizer setting to 0.68% at 1 liter per minute, maintaining the end-tidal concentration ≥0.5 MAC. The liquid agent consumed during the 28 minutes is 1.46 mL (Table 1).

As a control group (Simulation #3), we consider a "smart" anesthesia machine with end-tidal agent control. (In our opinion, this is the ideal solution because it makes the problem we study moot.) The anesthesia machine cannot become distracted (i.e., unlike an anesthetist with other responsibilities who can forget to turn the vaporizer back on, causing patient awareness during surgery [7]). The machine, instructed to achieve 1.0% (0.5 MAC), turns the vaporizer off while using 8 liters per minute of fresh gas flow. The end-tidal concentration of 1.0% is achieved in 0.9 minutes. Restarting the vaporizer at 0.68%, the 1 liter per minute flow rate was used for the remaining 27.1 minutes to achieve the same duration of 28 minutes. The estimated agent consumption is 1.01 mL. Therefore, the preceding approach (Simulation #2) of using a large 8 liter per minute fresh gas flow for 1.8 minutes resulted in an extra 0.45 mL sevoflurane used, where 0.45 mL=1.46 mL-1.01 mL. That estimate of 0.45 mL is our primary result.

We repeated Simulation #2 for an 80-year-old patient. An inspired concentration of 1.43% achieved end-tidal 1.39%, 1.0 MAC. During closure, the vaporizer was reduced to 0.43% (i.e., MAC-awake), with the fresh gas flow at 8 liters per minute. The time to achieve 0.70% (i.e., 0.5 MAC) was the same 1.8 minutes as for Simulation #2. Thus, our results are insensitive to the patient's age.

## Discussion

Our primary result was that to rapidly achieve the target of 0.5 MAC by the end of a long surgery, transient use of high fresh gas flow results in small (0.45 mL extra sevoflurane) increases in total anesthetic consumption. Such an approach is sufficient to reach the target more quickly than the duration of surgical closure for at least 95% of cases (Table 2). Applying the result, we consider whether many anesthetists would routinely make other decisions to reduce rare risks with a comparable environmental impact. The 0.45 mL of sevoflurane has a 100-year global warming potential of 73 gm carbon dioxide, where 73=0.45×(1.24 gm per mL)×130, and 130 is the unitless global warming potential ratio of for sevoflurane relative to carbon dioxide [26,27]. To avoid a rare but catastrophic event of intraoperative awareness [7,19,25], the carbon footprint cost is equivalent to the emission of the average US automobile driving 0.29 kilometers [28]. Based on a common driving speed limit of 40 kilometers (25 miles) per hour, a comparable decision would be to drive 26 seconds extra to avoid potential risk because 26=(0.29/(40 kilometers per hour))×(3600 seconds per hour). People regularly choose that decision (e.g., skip one mall exit without a traffic light and drive to the next one with such a device installed).

The environmental impact can appropriately be considered from the perspective of the cumulative effect over many cases. Thus, those above 73 gm of carbon dioxide would be multiplied by the number of related anesthetics. However, on balance then should be considered the total societal cost of the resulting prolonged times to tracheal extubation, including reintubation [3], respiratory treatments [3], dissatisfaction [4], longer operating room times that increase costs [5], and reduced productivity [5,6].

Among the N=29,626 cases in Table 2, the mean and standard deviation of the times for closure are 20.3 minutes and 18.3 minutes, respectively [8-10]. The coefficient of variation of 90% is massive. Although we could make the results more complicated by varying physiological values in Gas Man from its default parameters [11-13], conclusions would be unchanged because the dominating source of variability is not patients and physiology [16-18] but surgical procedures, surgeons, and cases [8-10,21-23]. Although we could make the results (vastly) more complicated by reporting durations of anesthesia too brief for steady state, conclusions would also be unchanged because that has not reduced the variability in times for closure (Table 2).

Our study has two limitations. First, the environmental consequence of sevoflurane would be moot if there were complete anesthetic agent capture in operating rooms [15]; however, that is not nearly achieved with contemporary technology [29]. Second, we did not consider the use of nitrous oxide because, when nitrous oxide is used with sevoflurane, the nitrous oxide contributes many-fold more to global warming than the sevoflurane [26], even when neglecting that five times the amount used needs to be delivered to hospitals because of leakage in pipeline networks [30].

## Conclusions

Prevention of prolonged extubations is important clinically and managerially. At the end of the surgery, during surgical closure, this objective is countered by running low fresh gas flows for their environmental benefit and avoiding turning off the vaporizer to prevent a rare but catastrophic awareness event. Simulations show that for long-duration surgical cases, and when not using an anesthesia machine with end-tidal agent control, generally, it is not feasible with sevoflurane to achieve reliably a suitable target (e.g., 0.5 MAC) at the end of surgery within the duration of surgical closure, because of the large variability in closure time. A suitable strategy is to turn the agent down but not the vaporizer off and turn up the fresh gas flow for <2 minutes. The environmental consequence is comparable to driving one block extra to avoid potential danger.
